# Serologic Surveillance for Orthoflaviviruses and Chikungunya Virus in Bats and Opossums in Chiapas, Mexico

**DOI:** 10.3390/v17050590

**Published:** 2025-04-22

**Authors:** J. Manuel Aranda-Coello, Carlos Machain-Williams, Manuel Weber, Alma R. Dzul Rosado, Tyler R. Simpkins, Bradley J. Blitvich

**Affiliations:** 1 Departamento de Conservación de la Biodiversidad, El Colegio de la Frontera Sur, Lerma 24500, Campeche, Mexico; manuel.aranda@posgrado.ecosur.mx (J.M.A.-C.); mweber@ecosur.mx (M.W.); 2 Estudios en Una Salud, Unidad Profesional Interdisciplinaria de Ingeniería Campus Palenque, Instituto Politécnico Nacional, Palenque 29960, Chiapas, Mexico; carmachain@gmail.com (C.M.-W.); adzul@ipn.mx (A.R.D.R.); 3 Department of Veterinary Microbiology and Preventive Medicine, College of Veterinary Medicine, Iowa State University, Ames, IA 50011, USA; trsimpk@iastate.edu

**Keywords:** alphavirus, bats, chikungunya virus, flavivirus, opossums, Zika virus

## Abstract

We performed serologic surveillance for selected arthropod-borne viruses (arboviruses) in bats and opossums in the Lacandona Rainforest, Chiapas, Mexico, in 2023–2024. Sera were collected from 94 bats of at least 15 species and 43 opossums of three species. The sera were assayed by the plaque reduction neutralization test (PRNT) for antibodies to eight orthoflaviviruses (dengue viruses 1–4, St. Louis encephalitis virus, T’Ho virus, West Nile virus, and Zika virus) and one alphavirus (chikungunya virus; CHIKV). Twelve (12.8%) bats and 15 (34.9%) opossums contained orthoflavivirus-specific antibodies. One bat (a Jamaican fruit bat) was seropositive for Zika virus, and 11 bats contained antibodies to an undetermined orthoflavivirus, as did the 15 opossums. All bats and most opossums seropositive for an undetermined orthoflavivirus had low PRNT titers, possibly because they had been infected with another (perhaps unrecognized) orthoflavivirus not included in the PRNTs. Antibodies that neutralized CHIKV were detected in three (7.0%) opossums and none of the bats. The three opossums had low CHIKV PRNT titers, and therefore, another alphavirus may have been responsible for the infections. In summary, we report serologic evidence of arbovirus infections in bats and opossums in Chiapas, Mexico.

## 1. Impacts

- Orthoflavivirus-specific antibodies were detected by the plaque reduction neutralization test in 12 of 94 (12.8%) bats in the Lacandona Rainforest, Chiapas, Mexico. One bat (a Jamaican fruit bat) was seropositive for Zika virus, while the other 11 bats were seropositive for an undetermined orthoflavivirus.
- Orthoflavivirus-specific antibodies were detected by the plaque reduction neutralization test in 15 of 43 (34.9%) opossums in the Lacandona Rainforest. All seropositive opossums had antibodies to an undetermined orthoflavivirus.
- Antibodies that neutralized chikungunya virus were detected in 15 (34.9%) opossums in the Lacandona Rainforest, but the titers were low, and thus, another alphavirus may have caused the infections.

## 2. Introduction

Arthropod-borne virus (arbovirus) is a non-taxonomic term used to describe a large group of diverse viruses transmitted to vertebrate hosts by infected hematophagous arthropods, such as mosquitoes and ticks. Many arboviruses are zoonotic and cause major disease outbreaks among humans, domestic animals, and wildlife [1,2,3]. Arboviruses belong to multiple genera, but some of the most important arboviruses, in terms of their impact on human and animal health, are classified within the genera *Alphavirus* (*Togaviridae*) and *Orthoflavivirus* (*Flaviviridae*).

Many orthoflaviviruses associated with human disease occur in Mexico, including all four serotypes of dengue virus (DENV1-4), St. Louis encephalitis virus (SLEV), West Nile virus (WNV), and Zika virus (ZIKV), all of which are mosquito-borne. DENV is responsible for >100,000 confirmed cases in Mexico each year, and the number of suspected cases is four- to five-fold higher [4]. The overall economic impact of DENV in Mexico is estimated to be >US$130 million annually [5]. SLEV and WNV have caused fatal neurological disease and ZIKV has been associated with congenital malformations and Guillain-Barré syndrome in Mexico [6,7,8,9,10]. T’Ho virus (THOV) is another orthoflavivirus present in Mexico [11,12]. THOV has not been directly linked to human disease, but its closest known relatives are human pathogens, and therefore, THOV could be an unrecognized cause of human disease.

Several mosquito-borne alphaviruses that cause human disease circulate in Mexico. A notable example is chikungunya virus (CHIKV), which causes an acute febrile illness often accompanied by debilitating arthralgia [13,14,15,16]. Other medically important alphaviruses in Mexico include eastern equine encephalitis virus (EEEV), Venezuelan equine encephalitis virus (VEEV), and western equine encephalitis virus (WEEV), all of which have been associated with fatal disease outcomes in humans [17,18,19,20].

Bats (order Chiroptera) are among the most important wildlife reservoirs of zoonotic viruses. These animals harbor numerous viruses, including some of the deadliest viruses to humans. Viruses isolated from bats include Hendra, Nipah, Marburg, and rabies viruses, along with viruses closely related to severe acute respiratory syndrome coronavirus-1 and -2 [21,22,23,24,25,26]. Bats have also been implicated as reservoir hosts of Ebola virus and Middle Eastern respiratory syndrome coronavirus [27,28]. Isolations of alphaviruses (e.g., VEEV) and orthoflaviviruses (e.g., Japanese encephalitis virus and SLEV) have been made from bats [29,30,31]. Serologic and molecular data suggests that other alphaviruses and orthoflaviviruses naturally infect bats [32].

Opossums are small- to medium-sized marsupials classified within the order Didelphimorphia. Unlike bats, opossums are not common sources of zoonotic viruses, although rabies virus and, more pertinent to this study, various arboviruses (SLEV, WEEV, and WNV) have been isolated from these animals [33,34,35,36]. Serologic and molecular data have provided evidence that other arboviruses naturally infect opossums [37,38,39]. For example, DENV4 RNA was detected in an opossum in French Guiana [40].

In this study, we serologically assayed bats and opossums in the Lacandona Rainforest of Chiapas for selected orthoflaviviruses (DENV1-4, SLEV, THOV, WNV, and ZIKV) and one alphavirus (CHIKV). All of these viruses occur in Mexico and are recognized human pathogens, except for THOV, which is a potential human pathogen. The purpose of the study is to increase our understanding of the contribution of wildlife in the ecology of arboviruses in Mexico.

## 3. Materials and Methods

### 3.1. Study Sites

**Figure 1:**
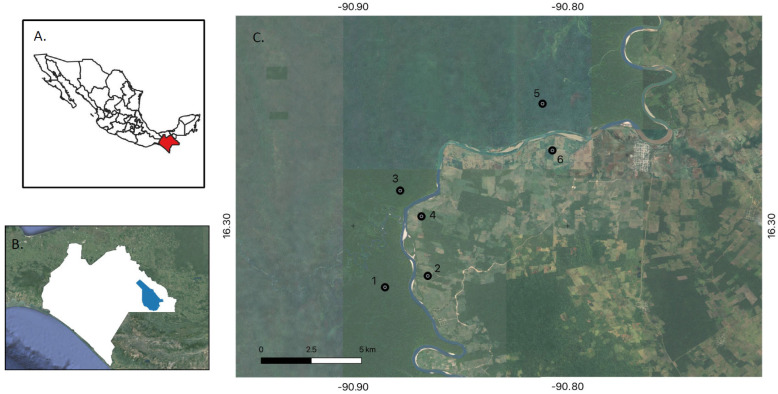
Geographic locations of the study area and sites, with (**A**) Chiapas shaded red, (**B**) Chiapas and the Lacandona Rainforest shaded white and blue, respectively, and (**C**) study sites denoted as black circles (designated 1 to 6) and longitudinal and latitudinal coordinates provided.

Study sites were established in the Lacandona Rainforest of Chiapas, southeast Mexico (Figure 1). Three study sites (denoted herein as undisturbed sites) were in the Montes Azules Biosphere Reserve, a protected area of the Lacandona Rainforest where human disturbance is minimal. Three other study sites (denoted herein as disturbed sites) were in the transition zone between forested areas and farmland in the ejido (communal land used primarily for agriculture) of Galacia, a small town in the municipality of Marqués de Comillas. Study sites were paired, with one undisturbed and one disturbed site in each pair. The two sites of each pair were 2 to 3 km apart from one another and separated by the Lacantún River. The paired sites were at 35-km intervals along the river.

### 3.2. Trapping of Bats

Trapping of bats was performed on 11 occasions: once each in May, June, August, September, and October 2023 and once each in January, March, April, June, July, and August 2024. Two mist nets (12 m in length, 3 m in height, 36 mm mesh openings) were set at each site. Nets were positioned at least 100 m apart and 1.5 m above the ground. Nets were open from 6:00 pm until midnight and checked for bats every 10 min. Captured bats were individually held in cloth bags until processing. Bats were physically inspected for signs of disease, weighed, and morphologically identified according to species, sex, and age class (juvenile, sub-adult, or adult) using published keys [41]. Red fingernail paint was used to mark the claws on the left leg of each bat to identify recaptures.

### 3.3. Trapping of Opossums

Trapping of opossums was performed on 11 occasions: once each in May, June, August, September, and October 2023 and once each in January, March, April, June, July, and August 2024. Four Tomahawk live-traps (81 × 25 × 30 cm; Tomahawk Live Trap Inc., Tomahawk, WI, USA) were used at each site. Traps were placed in a straight line at 500 m intervals along a central transect of 1.5 km. Traps were baited twice a day (8:00 am and 6:00 pm) using a mixture of tuna, eggs, apples, bananas, and oats to target a variety of opossum species. Traps were active for five consecutive days (24 h per day) and checked every eight hours. Captured opossums were chemically immobilized with Anesketin^®^, a ketamine-containing (100 mg/mL) solution (Pisa Farmaceutica, Atitalaquia, Hidalgo, Mexico) administered intramuscularly at a dose of 10 mg/kg [42]. Opossums were physically inspected for signs of disease and morphologically identified according to species, sex, and age class (juvenile, sub-adult, or adult) using published keys [43]. Red fingernail paint was used to mark the claws on the left leg of each opossum to identify recaptures.

### 3.4. Blood Collections

Whole blood was collected from bats by ulnar vein puncture and from opossums by non-lethal cardiac or tail vein puncture. The amount of blood taken from each animal did not exceed 1% of its estimated body weight (≤100 µL and 1.0 mL for bats and opossums, respectively). Blood samples were diluted 10-fold using field diluent (phosphate-buffered saline containing 0.75% bovine albumin fraction V, 500 units/mL penicillin, 500 μg/mL streptomycin, and 12.5 μg/mL amphotericin) and placed on ice. Animals were released alive immediately after blood collection. Samples were transported to the laboratory, centrifuged, and stored at −80 °C. All animals were handled and treated in accordance with the protocols reviewed and approved by the Institutional Animal Care and Use Committee of the El Colegio de la Frontera Sur, Lerma, Campeche, Mexico (Project No. CE-10-10-22).

### 3.5. Viruses

Nine viruses were used in this study: CHIKV (strain CH-R-1950), DENV-1 (strain Hawaii), DENV-2 (strain NGC), DENV-3 (strain H-87), DENV-4 (strain 241), SLEV (strain TBH-28), WNV (strain NY99-35261-11), ZIKV (strain PRVABC59) (Genbank Accession Nos. MG921596, KM204119, KM204118, P27915, AY947539, EU906867, AF196835, and MH158237, respectively), and a chimeric orthoflavivirus, designated ZIKV-THOV (prM-E), that contains the pre-membrane and envelope protein genes of T’Ho virus (Genbank Accession No. EU879061) in the genetic background of ZIKV. The isolate of CHIKV was originally recovered from a patient in northern Mexico [44]. Wild-type orthoflaviviruses were obtained from the World Health Organization Center for Arbovirus Reference and Research maintained at the Centers for Disease Control and Prevention, Division of Vector-Borne Infectious Diseases (Fort Collins, CO, USA). Construction of the chimeric orthoflavivirus is described elsewhere [45]. Viruses underwent no more than five passages in cell culture in our laboratories.

### 3.6. Plaque Reduction Neutralization Tests

Plaque reduction neutralization tests (PRNTs) were performed in six-well plates containing confluent monolayers of African green monkey kidney (Vero) cells (American Type Culture Collection, Manassas, VA, USA) following standard protocols [46] (Beaty, Calisher, & Shope, 1995). Sera were initially tested at a single dilution of 1:40 using DENV2 and WNV, two of the most widespread orthoflaviviruses in the Americas [47,48,49]. Titers were expressed as the reciprocal of serum dilutions yielding ≥70% reduction in the number of plaques (PRNT_70_). These assays are not DENV2- or WNV-specific; orthoflaviviruses are antigenically similar, and therefore, antibodies to other orthoflaviviruses are also detected [32]. All sera with antibodies that neutralized DENV2 or WNV were serially diluted using a starting dilution of 1:40 and tested by PRNT using all eight of the selected orthoflaviviruses. In these assays, titers were expressed as the reciprocal of serum dilutions yielding ≥90% reduction in the number of plaques (PRNT_90_). For etiologic diagnosis, the PRNT_90_ antibody titer to the respective virus was required to be at least four-fold greater than that to the other orthoflaviviruses tested. If neutralizing antibodies were detected but there was not at least a fourfold difference in PRNT_90_ antibody titers, the animal was considered to have antibodies to an undetermined orthoflavivirus. All sera were also tested by PRNT using CHIKV.

## 4. Results

### 4.1. Bat Collections

Ninety-four bats of ≥15 species were sampled (Table 1). The most highly represented species were the greater bulldog bat (*Noctilio leporinus*), greater sac-winged bat (*Saccopteryx bilineata*), and Seba’s short-tailed bat (*Carollia perspicillata*), comprising 16.0%, 11.7%, and 11.7%, respectively, of the bats collected. Fifty-two (55.3%) bats were male, and 42 bats (44.7%) were female. Adults (68.1%) were more common than sub-adults (20.2%) and juveniles (11.7%). Most bats (68.1%) were trapped at undisturbed study sites. Bats had a mean weight of 31.7g (with a standard deviation of 24.5 g). All bats appeared healthy.

### 4.2. Opossum Collections

Forty-three opossums of three species were sampled (Table 2). The most highly represented species was the gray four-eyed opossum (*Philander opossum* 41.9%), followed by the common opossum (*Didelphis marsupialis*; 32.6%), and Virginia opossum (*Didelphis virginiana*; 25.6%). Thirty-two (74.4%) opossums were male, and 11 opossums (25.6%) were female. Adults (46.5%) were more common than juveniles (41.9%) and sub-adults (11.6%). Most opossums (60.5%) were trapped at undisturbed study sites. All opossums appeared healthy.

### 4.3. Orthoflavivirus Serosurvey

Sera from all bats and opossums were screened at a dilution of 1:40 by PRNT_70_ using DENV2 and WNV. Twelve (12.8%) bats of seven species and 15 (34.9%) opossums of three species were revealed to contain orthoflavivirus-specific antibodies (Table 1, Table 2, Table 3 and Table 4). Of the bat species where >10 individuals were captured, the seroprevalence rate was highest (40.0%) for the Jamaican fruit bat (*Artibeus jamaicensis*). Of the opossum species where >10 individuals were captured, the seroprevalence rate was highest (44.4%) for the gray four-eyed opossum (*Philander opossum*). Antibodies to orthoflaviviruses were detected in bats and opossums of all life stages (juvenile, sub-adult, and adult), sexes, and habitats (disturbed and undisturbed) (Table 5). There were not statistically significance differences in seroprevalence according to life stage, sex, and habitat.

All sera with orthoflavivirus-specific antibodies were further diluted and tested by PRNT_90_ for antibodies to eight orthoflaviviruses. One bat (denoted as M96) was seropositive for ZIKV (Table 6). The bat was an adult Jamaican fruit bat trapped at an undisturbed study site. Eleven bats had antibodies to an undetermined orthoflavivirus(es), including one bat (M162) that was seropositive only when the less-stringent PRNT_70_ was used. Of the bats with antibodies to an undetermined orthoflavivirus(es), PRNT_90_ titers were usually highest for DENV1 or ZIKV, but most titers did not exceed 80. The 15 opossums also had antibodies to an undetermined orthoflavivirus (Table 7). Five opossums (T19, T24, T32, T33, and T35) were seropositive only when the less-stringent PRNT_70_ was used (Table 7). PRNT_90_ titers were usually highest for one of the DENV serotypes, but only one opossum (T31) had antibody titers that exceeded 160.

### 4.4. Chikungunya Virus Serosurvey

Sera from all bats and opossums were screened at a dilution of 1:40 by PRNT_70_ using CHIKV. None of the bats, but three (7.0%) opossums, were seropositive (Table 8). The antibodies were detected in two (11.1%) gray four-eyed opossums and one (9.1%) Virginia opossum. Using the more stringent PRNT_90_, none of the opossums were seropositive at a serum dilution of 1:40.

## 5. Discussion

We report the detection of orthoflavivirus-specific antibodies in bats and orthoflavivirus- and alphavirus-specific antibodies in opossums in the Lacandona Rainforest of Chiapas, Mexico. One Jamaican fruit bat was seropositive for ZIKV, but the viral species responsible for all other infections were not identified. Three Jamaican fruit bats in Yucatan, Mexico in 2022–2023 contained ZIKV RNA, as revealed by RT-PCR and Sanger sequencing [50] (Yeh-Gorocica et al., 2024). ZIKV RNA was also detected in two (9.1%) Jamaican fruit bats in Yucatan, Mexico in 2017 [51] (Torres-Castro et al., 2021). Jamaican fruit bats experimentally inoculated with ZIKV produced antibodies that reacted with viral antigen by enzyme-linked immunosorbent assay [52] (Malmlov et al., 2019). Some bats contained ZIKV RNA and antigen in selected tissues, but none had detectable viremia. Taken together, the aforementioned data suggest that Jamaican fruit bats are susceptible to both natural and experimental ZIKV infection but are unlikely to be reservoir hosts.

Eleven bats contained antibodies to an undetermined orthoflavivirus, and most had PRNT_90_ titers that did not exceed 80, which could be considered low. Others have reported similar findings [53,54,55,56,57] (Cui et al., 2008; Machain-Williams et al., 2013; Platt et al., 2000; Rucci et al., 2024; Stone et al., 2018). Twenty-six (18.6%) bats captured in Yucatan, Mexico, in 2010, and assayed by PRNT using all DENV serotypes, SLEV, and WNV contained orthoflavivirus-specific antibodies, but all were seropositive to an undetermined orthoflavivirus, and none had PRNT_90_ titers above 80 [54] (Machain-Williams et al., 2013). Fifteen (23.8%) bats in Costa Rica and Ecuador in 1998 contained antibodies that neutralized at least one serotype of DENV, but all PRNT_80_ titers were ≤80 [55] (Platt et al., 2000).

One explanation why the seropositive bats had low PRNT titers is the infections were caused by an orthoflavivirus(es) not included in our PRNTs. Orthoflaviviruses are closely related antigenically, and therefore, antibodies to one orthoflavivirus can cross-react with other orthoflaviviruses [58] (Rathore & St John, 2020). Orthoflaviviruses that infect bats and occur in the Americas include Montana myotis leukoencephalitis virus, Rio Bravo virus, and Tamana bat virus [59,60,61,62] (Bell and Thomas, 1964; Blitvich and Firth, 2017; Burns and Farinacci, 1956; Price, 1978). Alternatively, an unrecognized orthoflavivirus could have caused the infections. Because the bats were small, the serum volumes were insufficient to allow for the inclusion of additional viruses in the PRNTs.

The bats seropositive for an undetermined orthoflavivirus often had PRNT_90_ titers that were highest for DENV1 or ZIKV, although, as already noted, all were ≤80. Therefore, another explanation for the low PRNT titers is that DENV1 and ZIKV were responsible for some of the infections but replicate poorly in bats, resulting in low-level antibody production. Experimental infection studies have provided insight into the replicative abilities of DENV1, ZIKV, and other orthoflaviviruses in bats [52,63,64,65,66,67,68] (Aguilar-Setien et al., 2023; Cabrera-Romo et al., 2014; Davis et al., 2005; La Motte, 1958; Malmlov et al., 2019; Perea-Martinez et al., 2013; van den Hurk et al., 2009). All three Jamaican fruit bats held for 28 days after ZIKV inoculation developed ELISA antibody titers of 3200 [52] (Malmlov et al., 2019). In contrast, antibodies to ZIKV were not detected by PRNT_50_ in any of nine great fruit-eating bats (*Artibeus lituratus*) held for up to 21 days after inoculation [63] (Aguilar-Setien et al., 2023). All Jamaican fruit bats inoculated with DENV1 or DENV4 were negative by ELISA for DENV-reactive IgG [64] Two of 23 (8.7%) intermediate fruit-eating bats (*Artibeus intermedius*) inoculated with DENV2 and held for up to 23 days seroconverted [67] (Perea-Martinez et al., 2013). Differences in bat species, viral species or strains, time of serum collections, and antibody detection techniques could be why some bats contained detectable levels of orthoflavivirus-specific antibodies while others did not.

Fifteen (34.9%) opossums contained orthoflavivirus-specific antibodies. Evidence of orthoflavivirus (e.g., WNV) infection in opossums has previously been reported [69,70,71] (Blitvich, Juarez, Tucker, Rowley, and Platt, 2009; Gomez et al., 2008; Root et al., 2005). All seropositive opossums in our study contained antibodies to an undetermined orthoflavivirus, and often the PRNT_90_ titers were ≤80. These opossums could have been infected with another (perhaps unrecognized) orthoflavivirus not included in the PRNTs. Other orthoflaviviruses that occur in Latin America include Bussuquara, Cacipacoré, Ilhéus, Iguape, Rocio, and yellow fever viruses, although none have been isolated from humans, vertebrate animals or arthropods in Mexico [72,73] (Figueiredo, 2000; Rodriguez-Morales and Bonilla-Aldana, 2022). Further, none of the aforementioned viruses have been isolated from naturally infected opossums, although monotypic seroprevalence for Ilhéus virus was detected in two (16.7%) gray four-eyed opossums in Brazil [74] (Bernal et al., 2021).

As already noted, most opossums seropositive for an undetermined orthoflavivirus had low PRNT_90_ titers, and most of these titers were highest for one of the DENV serotypes. Therefore, some of the opossums seropositive for an undetermined orthoflavivirus could have been infected with DENV and the low PRNT titers could be the result of inefficient viral replication and a limited immune response. Several studies have investigated whether experimentally inoculated opossums support the replication of orthoflaviviruses (e.g., Powassan virus, SLEV, and ZIKV), but none were performed using DENV [75,76,77] (Kokernot, Radivojevic, and Anderson, 1969; McLean, Francy, and Campos, 1985; Thomas et al., 2023).

Surveillance studies have previously reported the presence of antibodies to CHIKV in bats [57,78,79] (de Souza, Gaye, et al., 2024; Kading et al., 2022; Stone et al., 2018). For example, 15 (36%) of 42 bats in Grenada, West Indies, in 2015 were seropositive for CHIKV [57] (Stone et al., 2018). Other surveillance studies have provided no serologic or molecular evidence of CHIKV infection in bats [80,81] (Bittar et al., 2018; Hernandez-Aguilar, Lorenzo, Ramirez-Palacios, Santos-Moreno, and Naranjo, 2023). A subset (50%) of big brown bats (*Eptesicus fuscus*) experimentally inoculated with a South African strain of CHIKV seroconverted at 14 DPI, while none of the bats inoculated with a CHIKV strain from the Comoros Islands seroconverted after the same length of time [82] (A. M. Bosco-Lauth, Nemeth, Kohler, and Bowen, 2016). CHIKV-reactive antibodies were not detected in any bats in our study. The absence of CHIKV-reactive antibodies could be because the incidence of CHIKV in the Americas has greatly decreased in recent years [78] (de Souza, Ribeiro, et al., 2024). Differences in the geographic locations of study areas, genetic compositions of circulating CHIKVs, and bat species could also explain why some studies provide evidence of CHIKV infection in bats while others did not. We did not include additional alphaviruses in the PRNTs because the sera volumes were too small, but previous studies have provided evidence that other alphaviruses naturally infect bats [73,83,84,85,86,87] (Barrantes Murillo et al., 2022; Fischer et al., 2021; Guzman, Calderon, Martinez, Oviedo, and Mattar, 2019; Kading et al., 2022; Moreira Marrero, Botto Nunez, Frabasile, and Delfraro, 2022; Thompson et al., 2015).

Three (7.0%) opossums contained CHIKV PRNT_70_ titers of 40. Our findings could indicate that the opossums had been infected with another alphavirus that occurs in Mexico (e.g., EEEV, VEEV or WEEV) [17,20] (Azar et al., 2020; Zacks and Paessler, 2010). Serum volumes were insufficient to allow for additional viruses in the PRNT. We also cannot dismiss the possibility that the opossums were exposed to CHIKV, but limited antibody production occurred. Opossums have never been experimentally inoculated with CHIKV, and, to the best of our knowledge, no other surveillance studies have reported the presence of CHIKV-reactive antibodies in these animals. However, surveillance studies have provided some insight into the susceptibility of opossums to other alphaviruses [88,89,90] (Bigler, Lassing, Buff, Lewis, and Hoff, 1975; Bigler et al., 1976; Estrada-Franco et al., 2004; Walder, Suarez, and Calisher, 1984). In one study, VEEV-reactive antibodies were detected in several gray four-eyed opossums and a common opossum in Chiapas, Mexico, in 2000–2001 [91] (Estrada-Franco et al., 2004).

One limitation of our study is that the initial orthoflavivirus PRNTs were conducted using only two viruses: DENV2 and WNV. As a result, we may have underestimated the true seroprevalence of orthoflaviviruses in bats and opossums within the study area. Additional seropositive animals may have been identified if the sera had been tested at a dilution of 1:40 against all eight selected orthoflaviviruses. Unfortunately, the limited serum volumes obtained from most animals precluded the inclusion of additional viruses in the initial PRNTs. We prioritized DENV2 and WNV because they are arguably the most common and geographically widespread Aedes-borne and Culex-borne orthoflaviviruses, respectively, in the Americas. It is also important to note that we used the more sensitive PRNT_70_ for the initial screening, which allows for the detection of lower amounts of neutralizing antibodies that could be missed with the more stringent PRNT_90_.

To summarize, we detected orthoflavivirus-specific antibodies in bats and orthoflavivirus- and alphavirus-specific antibodies in opossums in the Lacandona Rainforest of Chiapas, Mexico. One bat was seropositive for ZIKV, but the specific viruses responsible for all other infections were not determined. Additional surveillance studies are necessary to determine whether unrecognized vertebrate-infecting orthoflaviviruses and alphaviruses occur in Mexico, especially since many viruses from these taxonomic groups are zoonotic pathogens of humans.

## Figures and Tables

**Table 1:** Bats sampled in the Lacandona Rainforest, Chiapas, Mexico, in 2023–2024.

Species	Common Name	No. Sampled	No. (%) with Orthoflavivirus-Specific Antibodies
*Artibeus jamaicensis*	Jamaican fruit bat	10	4 (40.0)
*Artibeus lituratus*	Great fruit-eating bat	5	2 (40.0)
*Artibeus* spp.	-	1	0 (0)
*Carollia perspicillata*	Seba’s short-tailed bat	11	0 (0)
*Carollia sowelli*	Sowell’s short-tailed bat	7	0 (0)
*Dermanura watsoni*	Solitary fruit-eating bat	6	0 (0)
*Desmodus rotundus*	Common vampire bat	5	1 (20.0)
*Glossophaga mutica*	Merriam’s long-tongued bat	1	0 (0)
*Noctilio leporinus*	Greater bulldog bat	15	0 (0)
*Phyllostomus hastatus*	Greater spear-nosed bat	2	0 (0)
*Platyrrhinus helleri*	Heller’s broad-nosed bat	5	1 (20.0)
*Saccopteryx bilineata*	Greater sac-winged bat	11	2 (18.2)
*Sturnira parvidens*	Little yellow-shouldered Mesoamerican bat	5	1 (20.0)
*Trachops cirrhosus*	Fringe-lipped bat	6	1 (16.7)
*Uroderma bilobatum*	Tent-making bat	1	0 (0)
*Uroderma convexum*	Pacific tent-making bat	3	0 (0)
Total		94	12 (12.8)

**Table 2:** Opossums sampled in the Lacandona Rainforest, Chiapas, Mexico, in 2023–2024.

Species	Common Name	No. Sampled	No. (%) with Orthoflavivirus-Specific Antibodies
*Didelphis marsupialis*	Common opossum	14	5 (35.7)
*Didelphis virginiana*	Virginia opossum	11	2 (18.2)
*Philander opossum*	Gray four-eyed opossum	18	8 (44.4)
Total		43	15 (34.9)

**Table 3:** Bats with sera that neutralized dengue virus 2 or West Nile virus when screened by 70% plaque reduction neutralization test †.

Identification Number	Species	Collection Date (Day/Month/Year)	Study Site ‡	Age Class	Sex	PRNT_70_ Outcome	
						DENV2	WNV
M95	*Artibeus lituratus*	01/28/2024	3	Adult	F	+ §	− ¶
M96	*Artibeus jamaicensis*	01/28/2024	3	Adult	M	−	+
M104	*Sturnida parvidens*	03/28/2024	2	Sub-adult	F	−	+
M139	*Trachops cirrhosus*	07/30/2024	6	Adult	F	+	+
M141	*Saccopteryx bilineata*	07/30/2024	6	Adult	F	+	+
M142	*Saccopteryx bilineata*	07/30/2024	6	Juvenile	F	+	−
M147	*Desmodus rotundus*	07/30/2024	6	Adult	M	−	+
M158	*Artibeus jamaicensis*	08/01/2024	3	Adult	M	−	+
M162	*Platyrrhinus helleri*	08/01/2024	1	Sub-adult	M	−	+
M167	*Artibeus jamaicensis*	08/01/2024	1	Adult	M	−	+
M168	*Artibeus jamaicensis*	08/01/2024	5	Juvenile	M	−	+
M169	*Artibeus lituratus*	08/01/2024	5	Adult	F	−	+

† Sera were tested at a single dilution of 1:40; ‡ undisturbed and disturbed study sites are denoted by odd and even numbers, respectively; § positive; ¶ negative; DENV2, dengue virus 2; F, female; M, male; PRNT_70_, 70% plaque reduction neutralization test; WNV, West Nile virus.

**Table 4:** Opossums with sera that neutralized dengue virus 2 or West Nile virus when screened by 70% plaque reduction neutralization test †.

Identification Number	Species	Collection Date (Day/Month/Year)	Study Site ‡	Age Class	Sex	PRNT_70_ Outcome	
						DENV2	WNV
T17	*Philander opossum*	10/17/2023	4	Adult	F	+ §	+
T19	*Didelphis virginiana*	10/18/2023	3	Juvenile	F	− ¶	+
T24	*Philander opossum*	01/27/2024	6	Sub-adult	M	−	+
T25	*Didelphis virginiana*	01/28/2024	1	Adult	M	−	+
T26	*Philander opossum*	01/28/2024	5	Adult	M	+	+
T28	*Philander opossum*	01/29/2024	5	Adult	M	+	+
T29	*Philander opossum*	01/29/2024	5	Adult	F	−	+
T31	*Didelphis marsupialis*	01/29/2024	5	Adult	F	+	+
T32	*Philander opossum*	01/30/2024	2	Adult	M	−	+
T33	*Didelphis marsupialis*	01/30/2024	1	Adult	M	−	+
T35	*Didelphis marsupialis*	01/30/2024	2	Adult	M	−	+
T42	*Philander opossum*	03/28/2024	2	Juvenile	M	+	-
T44	*Didelphis marsupialis*	04/26/2024	3	Sub-adult	M	−	+
T53	*Didelphis marsupialis*	06/28/2024	4	Juvenile	M	−	+
T54	*Philander opossum*	07/30/2024	6	Adult	M	+	+

† Sera were tested at a single dilution of 1:40; ‡ undisturbed and disturbed study sites are denoted by odd and even numbers, respectively; § positive; ¶ negative; DENV2, dengue virus 2; F, female; M, male; PRNT_70_, 70% plaque reduction neutralization test; WNV, West Nile virus.

**Table 5:** Numbers and percentages of bats and opossums with orthoflavivirus-specific antibodies according to sex, life stage, and type of study site.

Description	No. Positive/Tested (%) for Orthoflavivirus-Specific Antibodies					
	Bats	χ²	*p*-Value	Opossums	χ²	*p*-Value
Sex						
Male	6/52 (11.5)			11/32 (34.4)		
Female	6/42 (14.3)	0.157	0.762	4/11 (36.4)	0.014	1.0
Life stage						
Juvenile	2/11 (18.1)			3/18 (16.7)		
Sub-adult	2/19 (10.5)			2/5 (40.0)		
Adult	8/64 (12.5)	0.636	0.793	10/20 (50.0)	4.77	0.086
Study site						
Disturbed	7/64 (10.9)			8/17 (47.1)		
Undisturbed	5/30 (16.7)	0.602	0.512	7/26 (26.9)	1.835	0.202

*p* < 0.05 is considered significance.

**Table 6:** Serologic summary of bats tested for antibodies to selected orthoflaviviruses by 90% plaque reduction neutralization test.

Identification Number	PRNT_90_ Titer								PRNT_90_ Outcome
	DENV1	DENV2	DENV3	DENV4	SLEV	THOV	WNV	ZIKV	
M95	-	- †	-	-	-	40	-	80	ORTHO
M96	-	-	-	-	40	40	-	160	ZIKV
M104	-	-	-	-	-	-	40	-	ORTHO
M139	40	40	-	80	-	80	40	80	ORTHO
M141	80	40	-	80	-	80	40	160	ORTHO
M142	-	80	-	40	-	40	-	40	ORTHO
M147	80	-	40	-	-	-	-	-	ORTHO
M158	80	-	40	-	-	-	-	-	ORTHO
M162	-	-	-	-	-	-	-	-	Negative
M167	40	-	-	-	-	-	-	-	ORTHO
M168	40	-	-	-	-	-	-	-	ORTHO
M169	40	-	-	40	-	-	-	40	ORTHO

† < 40; PRNT_90_, 90% plaque reduction neutralization test; DENV1, dengue virus 1; DENV2, dengue virus 2; DENV3, dengue virus 3; DENV4, dengue virus 4; ORTHO, undetermined orthoflavivirus; SLEV, St. Louis encephalitis virus; THOV, T’Ho virus; WNV, West Nile virus; ZIKV, Zika virus.

**Table 7:** Serologic summary of opossums tested for antibodies to selected orthoflaviviruses by 90% plaque reduction neutralization test.

Identification Number	PRNT_90_ Titer								PRNT_90_ Outcome
	DENV1	DENV2	DENV3	DENV4	SLEV	THOV	WNV	ZIKV	
T17	- †	-	-	40	-	-	-	-	ORTHO
T19	-	-	-	-	-	-	-	-	Negative
T24	-	-	-	-	-	-	-	-	Negative
T25	160	-	40	40	-	80	40	80	ORTHO
T26	-	-	-	-	-	40	-	-	ORTHO
T28	160	40	80	80	-	40	40	-	ORTHO
T29	-	-	-	-	-	-	40	-	ORTHO
T31	1280	640	320	640	320	640	320	160	ORTHO
T32	-	-	-	-	-	-	-	-	Negative
T33	-	-	-	-	-	-	-	-	Negative
T35	-	-	-	-	-	-	-	-	Negative
T42	-	40	-	-	-	-	-	-	ORTHO
T44	40	-	-	-	-	-	-	-	ORTHO
T53	-	-	-	-	-	-	-	-	Negative
T54	40	40	-	80	-	-	-	-	ORTHO

† < 40, PRNT_90_: 90% plaque reduction neutralization test, DENV1: dengue virus 1, DENV2: dengue virus 2, DENV3: dengue virus 3, DENV4: dengue virus 4, ORTHO: undetermined orthoflavivirus, SLEV: St. Louis encephalitis virus, THOV: T’Ho virus, WNV: West Nile virus, ZIKV: Zika virus

**Table 8:** Opossums with sera that neutralized chikungunya virus when screened by 70% plaque reduction neutralization test †.

Identification Number	Species	Collection Date (Day/Month/Year)	Study Site ‡	Age Class	Sex	PRNT_70_ Outcome
T25	*Didelphis virginiana*	01/28/2024	1	Adult	M	+ §
T27	*Philander opossum*	01/29/2024	5	Adult	M	+
T30	*Philander opossum*	01/29/2024	5	Juvenile	M	+

† Sera were tested at a single dilution of 1:40; ‡ undisturbed and disturbed study sites are denoted by odd and even numbers, respectively; § positive; M, male; PRNT_70_, 70% plaque reduction neutralization test.

## Data Availability

Additional data will be provided by the corresponding author upon request.
